# Agenda-setting in first sessions of business coaching—a focus on coaches’ practices to manage the agenda and establish the working alliance

**DOI:** 10.3389/fpsyg.2023.1232090

**Published:** 2023-10-09

**Authors:** Sabine Jautz, Eva-Maria Graf, Melanie Fleischhacker, Frédérick Dionne

**Affiliations:** ^1^Department of English, Faculty of Arts and Humanities, University of Siegen, Siegen, Germany; ^2^Department of English, Faculty of Humanities and Education, University of Klagenfurt, Klagenfurt, Austria

**Keywords:** agenda-setting, working alliance, business coaching, first sessions, social actions

## Abstract

**Introduction:**

Agenda-setting is a central communicative task for professionals and a joint activity of all participants particularly at the onset of helping interactions such as coaching. Agreeing on goal(s) and assigning tasks alongside establishing a trustful bond prepare the ground for the success of the interaction. The professional agent initiates and sets the agenda as part of their professional role and responsibility, i.e., based on their professional epistemic and deontic authority. Concurrently, by orienting to clients’ epistemic authority and by yielding power, control, and agency to clients to co-manage the ensuing interaction, agenda-setting is the first opportunity for client-centeredness, which is a central characteristic and success factor for the working alliance in coaching.

**Procedure and Methods:**

We take first steps in filling a research gap by providing a first analysis of the interactional unfolding of agenda-setting in coaching and by showcasing that and how agenda-setting as a joint activity of coach and client contributes to their working alliance. More precisely, we investigate agenda-management practices in five first sessions of business coaching to (1) document and analyze how the joint activity ‘agenda-setting’ is implemented via various (coach-initiated) social actions, (2) detail their contribution to establishing the working alliance, and (3) to interpret the emerging practices of agenda-management against the concept of ‘client-centeredness’. For the analysis, we draw on conceptual and methodological resources from interactional linguistics alongside linguistic pragmatics and conversation analysis.

**Results:**

We found 117 instances of ‘agenda-setting’ in our data which can be assigned to the seven social actions “Delivering Agenda Information”, “Requesting Agenda Information”, “Requesting Agenda Agreement”, “Requesting Agenda Action”, “Suggesting Agenda Action”, “Offering Agenda Action” and “Proposing Agenda Action”.

**Discussion:**

The social actions display that agenda-setting serves to establish a common ground regarding goals, tasks and the relational bond of coach and client, and (after this has been achieved) to negotiate future coaching actions. Thus, the joint activity of ‘doing’ agenda-setting can be shown to be ‘doing’ working alliance at the same time.

## 1. Introduction

“Accompanied development processes succeed when people know what is to be achieved, they are clear about what needs to be done to achieve it, and they feel confident enough to dare to initiate necessary change steps.” (Ehrenthal et al., 2020, p. 488; translated by SJ et al.)

Agenda-setting is a primary means and method to successfully establish and manage the working alliance in coaching, i.e., to agree on the goal(s) of the encounter, to assign the tasks to achieve these goals and to establish a trustful bond between coach and client. Insights into the interactional trajectory of agenda management offer an important perspective on the concrete local actions of coach and client and on how the working alliance is discursively achieved (Horvath and Muntigl, 2018 for psychotherapy). More globally, in the form of supra-session courses of actions (Bercelli et al., 2013), this underlies the successful transformation of relations, emotions and referents through sequentially structured practices along entire coaching sessions and processes (Peräkylä, 2019). While managing the agenda^1^ is a joint activity for coach and client along the entire coaching, it is particularly relevant at its onset, i.e., in first sessions. Agenda-setting prepares the common ground, i.e., “the sum of their [coaches’ and clients’; SJ et al.] mutual, common, or joint knowledge, beliefs, and suppositions” (Clark, 1996, p. 93) as the *sine qua non* for everything coach and client do. As such, it strongly influences the unfolding character of the interaction (Svinhufvud and Vehviläinen, 2013, p. 144; Graf and Jautz, 2022, p. 173). The interactional trajectory of agenda-setting and management (in first sessions) in coaching is closely interwoven with the domains of knowledge as well as power, control, and agency of the participants and how these are locally negotiated within the overall social organization of coaching. As Stevanovic et al. (2022, p. 2) argue,

(a)chieving equal participation in an encounter with two or more participants is always a great challenge (…). While this is the case even in dyadic encounters that are permeated by strong expectations of equality (…), the situation is naturally even more challenging in encounters in which participants have distinct roles and hierarchical statuses (…).

These distinct roles and hierarchical statuses in professional (helping) encounters such as coaching are tied to the participants’ differing epistemic and deontic authorities (Graf, 2019). Coaches’ territories of knowledge (Kamio, 1997) and experience (Heritage, 2011) cover their professional theories and expertise and center on structuring and guiding the process; clients’ territories of experience and knowledge cover their life-world perspective and center on the content of coaching (Deplazes et al., 2018; Graf, 2019). Coaches have specific social and interactional entitlements to impose (future) actions on their clients due to their professional role and hierarchical status as coaching (process) experts. Of particular interest, therefore, is how coach and client locally negotiate these entitlements to know and to impose (future) actions regarding the coaching agenda, i.e., what topics to cover and how to process them to allow for clients’ learning and change as the underlying goals of coaching. This ‘how’ is socio-culturally and institutionally framed by the conceptualization of coaching as professional interaction which is not expert-oriented, but client-oriented (Schein, 1978; Schreyögg, 2012). On a broader basis, such a fostering and promoting of shared power and responsibility between coach and client falls under the concept of ‘client-centeredness’ or ‘client participation’: Client-centeredness encompasses a relatively recent paradigmatic shift in the delivery of social and health care services and, more specifically, in the relationship between professional experts and clients: Following the definition by Stevanovic et al. (2022, p. 1), client-centeredness or client participation are conceived here not only as involving clients in deciding on their treatment (in medical encounters; see Robinson and Heritage, 2016), but more generally as clients’ right to influence the planning and development of the professional service.

Analyses of the local interactional unfolding of agenda-setting are so far missing in (linguistic) coaching process research (Fleischhacker and Graf, accepted for publication). The current paper addresses this research gap. We carry out a data-driven, inductive and exploratory study that investigates agenda-setting as interactional and discursive achievement in business coaching. We draw on five first sessions from a larger corpus of coaching interactions that was recently collected within the framework of the international and interdisciplinary research project *Questioning Sequences in Coaching* ^2^ (QueSCo, 2023). We pursue the following goals with our contribution: (1) document and analyze how the joint activity ‘agenda-setting’ is implemented via various (coach-initiated) social actions, (2) detail their contribution to establishing the working alliance, and (3) to interpret the emerging practices of agenda-management against the concept of ‘client-centeredness.’ As the purpose of this study is to give a first overview of the activity of agenda-setting in coaching, no in-depth micro-level analyses are carried out at this point.

## 2. Working alliance and agenda-setting

As outcome research across various professional contexts has convincingly illustrated, agreeing on goal(s) and task(s) alongside establishing a trustful bond between the participants prepare the ground for the overall success of the interaction. That is, setting and managing the agenda contributes to (initiating) the working alliance of coach and client, which in turn positively influences the overall success of the encounter. Agenda-setting thereby also represents the first and pivotal opportunity to locally act out client-centeredness (Gafaranga and Britten, 2003; Frankel et al., 2013; Gobat et al., 2015). In what follows, relevant aspects of both concepts for the current analysis will be detailed.

### 2.1. Working alliance

While building and managing relationships are part and parcel of all social interactions, in helping professions, the relationship between professionals and their patients/clients has proven central for the interactive construction of the process and for achieving the respective institutional tasks and goals (e.g., Miller and Considine, 2009; Horvath and Muntigl, 2018; Graf et al., 2019; Scarvaglieri, 2020; Scarvaglieri et al., 2022). The concept ‘therapeutic alliance’ or ‘working alliance’ (introduced by Greenson, 1965 and at times used synonymously; Horvath and Luborsky, 1993, p. 561), originated in (research on) psychodynamics. Nowadays, they find a pan-theoretical application to helping professions in general (Bordin, 1979; Horvath and Luborsky, 1993; Ackerman and Hilsenroth, 2003). Besides this broadening of its scope, what is of particular relevance for the current focus is the specification introduced by Bordin (1979, p. 252) of (agreement on) goal(s) of treatment, assignment of task(s) and the development of a bond as the three core components of the working alliance. The participants need to negotiate and agree on the overall goal(s) of the interaction, i.e., on clients’ concern(s) or goal(s) and coaches’ overall orientation to professional theories of change (Deplazes et al., 2018). They need to negotiate and agree on the relevant tasks to achieve the goals (Muntigl et al., 2020 on chair work in psychotherapy). And, finally, they need to build and maintain a trustful bond, which allows clients to open up, engage with the professional procedures and comply with the measures agreed on Scarvaglieri et al. (2022). Agenda-setting thus to a great extent underlies the working alliance and, at the same time, enables it.

In quantitative outcome research on psychotherapy, the therapeutic/working alliance represents an established and verified success factor (e.g., Horvath, 2006; Norcross and Lambert, 2018; Spencer et al., 2019; Wampold and Flückiger, 2023): “The strength of the alliance is arguably the best and most reliable predictor of outcomes […] and is generally considered one of the most important common factors in therapy” (Ribeiro et al., 2013, p. 295). And more specifically regarding the components of the working alliance, Muntigl et al. (2020, p. 2) argue that “(t)here is an accumulation of evidence that therapists and clients who can agree on the importance of the in-therapy activity proposed by the therapist, and actively collaborate in these tasks, have more successful outcomes than those who struggle to achieve such consensus.”

The significance of the working alliance for coaching success has more recently also been established in quantitative psychological outcome research (Baron et al., 2011; Behrendt, 2012; de Haan et al., 2016; Graßmann et al., 2019). Despite such empirical proof, Ianiro et al. (2013, p. 26) and others argue that “(a)ll in all, little is known of the interaction between coach and client and the interpersonal dynamics that constitute a high quality coaching relationship, although this is a matter of high interest for practitioners and researchers.” Such lack of insights into the concrete interactional practices of ‘doing the working alliance’ by coach and client resonates more generally with the continuing research gap regarding its locally ensuing verbal and non-verbal management by the participants across a variety of helping contexts (but see, e.g., Muntigl and Horvath, 2014; Muntigl et al., 2020; Scarvaglieri, 2020 for therapy; or Thurnherr, 2022 for counseling).

### 2.2. Agenda-setting

Steering the conversation is an omnipresent activity of participants in talk-in-interaction. Communicative partners prospectively and retrospectively control or influence the overall organization of the ensuing conversation via, e.g., turn-taking and turn design, the thematic development via introducing new topics or shifting topics and, more generally, the overall progressivity of joint actions and activities (for a detailed discussion see Tiittula, 2001; and for coaching Winkler, 2017).

While asymmetry, dominance, power, and hierarchy (e.g., Tiittula, 2001; Brock and Meer, 2004) are locally negotiated in any kind of conversations, this process has particular interactional consequences in professional and institutional contexts. Professional and institutional interaction is inherently goal- and task-oriented and the differing roles and responsibilities of the professional experts and clients/patients, alongside their knowledge and power with respect to these tasks and goals, is made relevant differently to serve this purpose (Drew and Heritage, 1992; Tiittula, 2001; Freed, 2015). Concurrently, neither power, knowledge, participants’ roles and identities nor the overall participation framework are (strictly) preordained notions (Sarangi, 2001; Gülich, 2003; Koester, 2010). Instead, they are locally (re-)negotiated and co-constructed in communicative loops alongside the encounter and show in the “momentary relationship of the participants” (Stevanovic and Peräkylä, 2014, p. 186) and the ensuing epistemic, deontic and affective orders. The same holds for agenda-setting as a crucial joint activity where tasks and goals are defined and negotiated in accordance with the professionals’ and clients’/patients’ roles, responsibilities, knowledge, and power. The overall thematic, interactional, and relational organization of the encounter is established in communicative loops throughout the sessions/process.

Research on agenda-setting (with a focus on helping interactions) is prolific for medical encounters, especially in physician-patient consultations. Agenda-setting is defined as a communicative strategy that physicians use at the beginning of clinical visits to elicit patients’ topics or concerns, to propose their own topics and to organize a list of shared topics (Boyd and Heritage, 2006). Yet, establishing the topical focus of a physical consultation presents a challenge, given that such time-limited encounters often involve multiple, interrelated priorities that need to be addressed (Gobat et al., 2015, p. 822). Effective agenda negotiation builds, following Manning and Ray (2002, p. 462), on a joint accomplishment by physician and patient, whose interaction “shows they are satisfactorily addressing each other’s concerns.” Extensive (conversation-analytic) research investigated clinicians’ (more or less effective) openings of medical visits (see, e.g., various publications by Heritage and Robinson).

Beyond this relatively narrow reading of agenda-setting as collecting and prioritizing relevant concerns during the problem presentation phase in medical encounters, e.g., Gobat et al. (2015) discuss a broader conceptualization: “agenda setting involved a process whereby patients and clinicians co-establish a joint focus for both their conversation and their working relationship” (p. 822). Beyond the topic-oriented domains of patients’ and clinicians’ concerns, agenda-setting also includes ‘agreement of shared priorities,’ ‘establishing conversational focus’ as well as ‘collaboration and engagement,’ all of which focus more on relational aspects. Agenda-setting as joint activity is also taken beyond opening sequences: “(a)genda setting is often used at the start of a clinical encounter, but can be used at any stage (…) (e.g., for realignment)” (Gobat et al., 2015, p. 825). It is necessarily flexible, as unexpected topics may arise in the conversation to which practitioners need to be responsive by revising the set agenda. Particularly in this respect, meta-communication and structuring activities are reported as essential parts of agenda-setting (Gobat et al., 2015, p. 824). Agenda-setting, in this broader sense, is understood as a process that allows practitioners and patients to align in three areas (Gobat et al., 2015, p. 825) that also underlie the working alliance: (a) the content of what will be discussed in the session (task), (b) the overall course of their work together, i.e., what both parties hope to achieve (goal), and (c) the relational ‘ground rules,’ e.g., who will adopt what kind of role and responsibilities (bond). In this sense, ‘(a)genda setting offers potential for clinicians and patients to collaborate more effectively in decision-making about their care’ (Gobat et al., 2015, p. 822). Such shared decision-making or ‘consensus-based’ decisions have received much empirical attention (see, e.g., Muntigl et al., 2020) as one result or consequence of client-centered agenda-setting, both in medical and psychiatric visits (Frankel et al., 2013, p. 195). In agenda-setting, clients’ epistemic authority over their subjective life experiences and their deontic authority to participate in decisions regarding the thematic and interactional trajectories of the professional encounter alongside co-conceptualizing the ensuing professional relationship are honored more generally.

Overall, agenda-setting in helping interactions fulfills a principal organizational, thematic, as well as relational function for the ensuing professional encounter with special relevance at its beginning, i.e., during first sessions. It is the professionals’ responsibility to organize the encounter following a more or less predetermined structure (see, e.g., Heritage and Maynard, 2006 for the physical consultations or Deplazes et al., 2018 for coaching). In turn, it is the thematic component of agenda-setting where patients/clients should have a decisive say. Yet, when and how this ‘space’ is given is often determined by the professional expert: “To use the time available effectively, to cover all the tasks, and to encourage talk about issues that usually are difficult to address (…), it is useful for the counselor to take initiatory actions and to control the agenda” (Peräkylä, 1995, p. 97). More generally, it can be argued that professionals’ interactional dominance is an institution-endemic, functional, and vital part of the encounter, something that also the clients endorse in and through their own conduct (Nanouri et al., 2022; Stevanovic et al., 2022, p. 1). It is particularly the overall ‘how’ of both the structural and the thematic agenda-setting that determines the relational quality of agenda-setting and, consequently, of the entire encounter: Alongside professional or client-controlled approaches to setting the agenda (Schein, 1978), “(…) agendas can be set collaboratively with each party contributing ideas about what is important to cover in the visit and negotiating whether and when these ideas will be discussed. This style of agenda-setting comes closest to being consumer-centered because it is based on shared power and control” (Frankel et al., 2013, p. 197).

In its micro-linguistic/interactional focus on agenda-setting, CA-based research on medical (see above), educational (e.g., Stephenson, 2020) and more “quasi-conversational” professional (helping) interactions such as counseling (e.g., Peräkylä, 1995; Vehviläinen, 2003) outlines agenda-setting as follows: “By agenda management we refer to the interactional moves in and through which a participant steers the topic of conversation, launches transitions and key shifts in the participants’ activity, and implements (…) actions, such as announcing decisions” (Stevanovic et al., 2022, p. 2). Agenda-setting establishes the common ground for the participants’ actions and activities and thus strongly influences the unfolding character of the coaching interaction (Svinhufvud and Vehviläinen, 2013, p. 144; Graf and Jautz, 2022, p. 173). Across all professional contexts, questioning practices are “the most typical way [for the professionals, SJ et al.] to manage the agenda” (Vehviläinen, 2003, p. 88). While agenda-setting questions are a primary tool in coaching, too (Fleischhacker et al., in prep), the current analysis seeks to go beyond social actions such as requesting information or agreement.

## 3. Data and methodology

### 3.1. Data

For the current study, we use five randomly selected first sessions from a recently collected corpus of work-related coaching processes from Germany and Switzerland. The coaching processes, both face-to-face and online, were carried out in German and were video- and audio-recorded by the coaches themselves. Coaches (in four sessions females, in one a male coach) are seasoned practitioners working in the realm of solution-oriented, systemic coaching; the clients (all female) had either an academic or an organizational background. The sessions were transcribed according to (simplified) CA transcription conventions (such as outlined, e.g., by Jefferson, 2004). For the current purpose, examples are translated into English. Original data can be found as Supplementary material. Written informed consent was obtained from all participants for the publication of anonymized data. Persons, organizations, places etc. referred to within the coaching, including names of coaches and clients have been replaced (see QueSCo, 2023 for more information).

### 3.2. Methods

We carry out a data-driven, inductive and exploratory research to understand how the joint activity ‘agenda-setting’ is managed by coaches (and clients) in first sessions of coaching. To this end, we use conceptual and methodological resources from linguistic pragmatics, interactional linguistics, and conversation analysis. From linguistic pragmatics (Clark, 1996), we adopt the overall action approach to language that considers language use as arising in joint activities, based on the coordinated actions of the participants, and the concept of ‘common ground’ as accumulating in joint activities, i.e., the participants’ shared knowledge, beliefs and suppositions about the action(s) at hand. From interactional linguistics (Couper-Kuhlen, 2014; Couper-Kuhlen and Selting, 2018) we draw on the concepts of ‘social action’ and ‘practices.’ We focus on how interactants implement social actions, i.e., actions produced and responded to in the ensuing interaction at hand (Couper-Kuhlen and Selting, 2018, p. 214), as part of a joint activity via recurrent form-based and content-based uses of language, i.e., practices (Couper-Kuhlen and Selting, 2018, p. 29). Finally, from conversation analysis we apply the basic conceptualization of interactions as being sequentially organized both in their thematic as well as their structural layout (Schegloff, 2007; Sidnell, 2010). In addition, the interwoven CA-based concepts of epistemics, i.e., participants’ authority based on knowledge and expertise, and of deontics, i.e., participants’ authority and power to determine future courses of actions, are drawn upon (Heritage, 2012, 2013; Stevanovic and Peräkylä, 2014; Stevanovic et al., 2022). While participants’ epistemic and deontic authority based on their social roles and identities form the background for action formation and ascription, their respective epistemic and deontic stances, i.e., their interactional displays of knowing and power, may make them appear more or less knowledgeable or powerful than they actually are or than their position in the social structure allows them to be.

### 3.3. Procedure

Initially motivated by agenda-setting questions as established in the *QueSCo* project, authors 1 and 2 examined all five first sessions for the occurrence of agenda management by coaches and clients. Beyond the narrower category of agenda-setting questions, 127 instances of agenda management, 117 initiated by coaches and 10 initiated by clients, were identified and further processed. In an iterative process, seven categories were established according to the types of social actions implemented by coaches that ‘do agenda management’: “(t)he particular sense of *action* being put central here is the ascription or assignment of a ‘main job’ that the turn is performing. The sense of ‘main job’ or primary action intended here is *what the response must deal with in order to count as an adequate next turn*” (Levinson, 2013, p. 107; emphasis in original). Social actions are additionally defined, following Couper-Kuhlen and Selting (2018, pp. 216f.), according to their turn design as well as their sequential position or placement within the coaching conversation. While we also consider the sequential organization of the social actions under scrutiny and thereby address action formation alongside action ascription, i.e., the (re-)definition of the interaction partners’ reaction to the social action, our primary focus is on coaches’ initiatory turns. In line with the socio-interactional layout of coaching as professional and institutional encounter, the vast majority of agenda moves (viz., 117 instances) is made by the coaches. Due to space limitations, we will not further discuss the 10 instances of client-initiated agenda-setting which we found in the corpus (but see Graf et al., in prep).

As regards turn design, we paid attention to aspects such as “subjecthood (you or me as agent?), interrogativity (are you asking me or telling me?), conditionality (is this a hypothetical [*sic*] or not?), modality (ability, willingness or necessity?) and imperativity (is non-compliance an option or not?)” (Couper-Kuhlen, 2014, pp. 640f.), which can form the basis for determining “favorite, or ‘preferred’ formats” (Couper-Kuhlen, 2014, p. 639) for the different social actions.

According to Stevanovic and Peräkylä (2014, p. 187), “(i)t seems as if the main difference between the major classes of social action would be related to the particular facet of the participants’ momentary relationship that each class makes relevant.” Relevant for the current analysis is—on the one hand—the respective epistemic status of the participants, i.e., coaches’ and clients’ [K+] or [K-] status (Heritage, 2012) with regard to agenda-/coaching-relevant information. Concurrently, “(i)n the process of action formation, nothing is more fundamental than determining whether an utterance is delivering information or requesting it” (Heritage, 2013, p. 557). Actions of delivering or requesting news or informing are thereby reserved for those utterances that are specifically designed to report something newsworthy or informative to the recipient (Couper-Kuhlen and Selting, 2018, p. 266) or to enquire about something newsworthy or informative for the speaker; in our case primarily information regarding the overall framing of coaching and clients’ issues or concerns. This is reflected in the categories “Delivering Agenda Information,” “Requesting Agenda Information,” and “Requesting Agenda Agreement” (see Table 1).

**Table 1:** (Re-)Actions related to agenda-/coaching-relevant information/knowledge status.

Coach’s action	[K+]	[K−]	Client’s socially preferred reaction
Delivering agenda-/coaching-relevant information	Coach	Client	Acknowledging information
Requesting agenda-/coaching-relevant information/agreement	Client	Coach	Providing information/agreement (confirmation)

On the other hand, the question of agent and beneficiary of coaching agenda-related future action determined our categorization. More generally, it focused on the participants’ rights to direct future actions (based on their (upgraded) epistemic status) (Stevanovic and Peräkylä, 2012; Stevanovic and Svennevig, 2015). In the context of requests for actions, Stevanovic and Peräkylä (2014, p. 192) argue that

(r)equests for action may range from orders and commands to suggestions and hints, depending most fundamentally on the extent that the first speaker may assume that the second speaker will perform the relevant action without being directly asked for it (…). Hence (…) we argue that such an interpretation is contingent on the recipient’s judgments about the speaker’s high deontic status relative to the recipient in the domain in question.

Based on Couper-Kuhlen’s (2014) classification, we categorized the remaining instances of agenda-setting practices into “Requesting Agenda Action,” “Suggesting Agenda Action,” “Offering Agenda Action” and “Proposing Agenda Action.” These actions focusing on agent and beneficiary of the (future, coaching-relevant) social action refer primarily, but not exclusively, to the negotiation of interventions (Table 2). As such, these actions entail a varying element of control as they influence the future activities of the interlocutors (Couper-Kuhlen and Selting, 2018, p. 259). How much control can be executed (also) shows in the linguistic practices that realize these social actions: “[T]he degree of entitlement to direct another’s actions (e.g., assigning homework; giving advice concerning a problem) is often realized in the linguistic design of the directive, such as whether imperative or declarative formats or whether certain modality markers (e.g., will, would, could, should, etc.) are used (…)” (Muntigl et al., 2020, p. 2).

**Table 2:** Distinctive dimensions of social actions (adapted from Couper-Kuhlen, 2014).

Social action	Agent of future action	Beneficiary of future action	Socially preferred reaction
Request	Other (client)	Self (coach)	Granting the requested action
Suggestion	Other (client)	Other (client)	Accepting the suggested action
Offer	Self (coach)	Other (client)	Accepting the offered action
Proposal	Self and other (coach and client)	Self and other (coach and client)	Agreeing with the proposed action

For each (sub-)category (see Table 3) a representative example was chosen for a detailed analysis. Besides categorizing the agenda management practices, the analysis also focused on whether the classified instances referred to the goal-, task- or bond-component of the working alliance; these components were assessed based on the thematic focus of the proposition. The linguistic turn design of the social actions was analyzed as regarding (repetitive) grammatical, lexical or syntactic features. Finally, epistemic and deontic stance taking was documented.

**Table 3:** Overview of agenda actions by coaches.

Agenda actions by coaches	Frequency
Delivering agenda information	36
*1.1 Structuring content/session/process/coaching*	*21*
*1.2 Commenting on own action*	*15*
Requesting agenda information	14
*2.1 Defining content/goal*	*11*
*2.2 Defining roles and responsibilities*	*3*
Requesting agenda agreement	15
Requesting agenda action	3
Suggesting agenda action	34
Offering agenda action	9
Proposing agenda action	6
Total	117

The categorization of the instances into seven social actions was critically discussed with authors 3 and 4, who also substantially contributed to the detailed analysis of the chosen examples and the interpretation of the findings. The degree of detailedness is dependent on the overall analytic goal, i.e., to give a first overview of agenda-setting practices in coaching. While the overall approach in this paper is qualitative in nature, the raw frequencies of the social actions and their respective sub-types were considered for the purpose of interpretation.

## 4. Analysis of agenda-management practices in first sessions of coaching

Table 3 provides an overview of the different types of coaches’ social actions alongside their sub-types as well as the frequencies of occurrence as found in the data. The categories are organized according to an interaction and content-based logic. Coaching relevant knowledge concerning content as well as the process must first be gathered from and negotiated by coach and client for both participants to upgrade their respective epistemic status, before future coaching-relevant agenda actions can be implemented. Even though this is not a strict order of social actions, it turns out to be a recurring pattern (across and within processes). In particular, agenda-setting in coaching is managed (by coaches) via the social actions “Delivering Agenda Information” [with the two subtypes “Structuring content/session/process/coaching” and “Commenting on own action” (see chapter 4.1)], “Requesting Agenda Information” [with the two subtypes “Defining content/goal” and “Defining roles and responsibilities” (see chapter 4.2)], and “Requesting Agenda Agreement” (see chapter 4.3), as well as the agenda action-related categories “Requesting Agenda Action,” “Suggesting Agenda Action,” “Offering Agenda Action” and “Proposing Agenda Action” (see chapters 4.4–4.7).

### 4.1. Delivering agenda information (*n* = 36)

A central part of setting and managing the agenda in coaching is informing clients about the overall organization of the interaction, i.e., setting up the interaction frame of ‘coaching’ regarding its content(s) as well as its temporal and structural layout. The primary communicative practice in the context of framing coaching methodologically, procedurally, and temporally (Graf, 2019) are ‘informing sequences’ or ‘informings’ (Schegloff, 2007; Thompson et al., 2015; Couper-Kuhlen and Selting, 2018). As supported by Silverman (1997), the coach as the professional agent has both the epistemic authority and status (and the deontic authority and status) to deliver information relevant for the coaching agenda to clients.

We found two different subtypes of delivering agenda information: Those that inform about future coaching steps, viz. how to structure the content, the session or process, or the coaching in general (4.1.1) and those which inform about coaches’ upcoming own actions (4.1.2). Delivering agenda information often entails information about time and place along with structuring devices to clarify what happens when. Coaches almost exclusively use declaratives, often phrased with first-person singular present tense forms. We find various uses of the indicative, but also conditional *would*, which renders the information delivery more polite and pays tribute to the clients’ negative face needs by granting more freedom of action (Brown and Levinson, 1987, pp. 129ff.). In the vast majority of cases, these agenda moves support transparency, thus contributing to establishing the bond between coach and client.

#### 4.1.1. Structuring content/session/process/coaching (*n* = 21)

21 out of the 36 examples of “Delivering Agenda Information” in our corpus have a structuring function. In providing structural information for clients and making the procedure etc. transparent, these agenda moves promote a trustful bond between coach and client; they also prepare for an agreement on the tasks to be carried out. Except for one imperative, all information deliveries are realized as declaratives, featuring predominantly first-person singular *I*, but also a few first-person plural *we* pronouns in subject position. Most of these examples contain temporal or spatial deixis (*at this point, here, just, later, hour, now, next session/time, time horizon, during, in the course of, takes more time, start, X hours of time, date, today*) and other structuring devices (*to make a point here*). When it comes to planning the future, the agenda moves also contain visual lexis (*look at, illuminate, clarity*). Examples often contain conditional forms to downgrade the coaches’ deontic claims. Example 1 illustrates this category.

**Example 1:** Delivering Agenda Information: Structuring content/session/process/coaching.


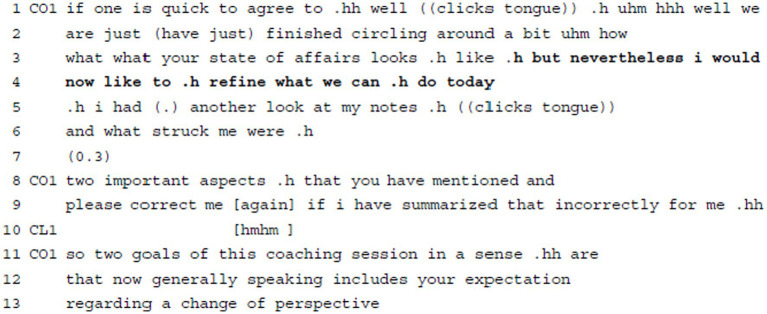


To arrive at a goal definition for this first session as a basis for working on the client’s issues in a narrow sense, the coach starts by summarizing what they have already done in the first couple of minutes of the session, i.e., that they have encircled the client’s concern more generally (“.h uhm hhh well we are we just (have just) finished circling around a bit uhm how what what your state of affairs looks .h like,” ll.1–3). The coach’s summary is characterized by various hesitation markers (“uhm,” “well,” l.1), audible breathing, a cut-off, self-repairs, and repetitions (“we are just (have just),” ll.1–2; “what what,” l.3), as well as modal particles (“a bit,” l.2) and rather unspecific vocabulary (“your state of affairs,” l.3). Addressing their prior work serves as a preparation and accounting for the coach’s attempt to define the goal of the current session. The latter is introduced via a contrastive “but nevertheless” (l.3), implying—together with the hesitant summary of their prior actions, the focus on the here-and-now (“now,” l.4)—that what they have been doing so far is insufficient regarding a goal definition. This leads the coach to formulate her wish to specify (“refine,” l.4; in contrast to “circling,” l.2) today’s goal or, more precisely, “what we can .h do today” (l.4), with the modal verb “can” denoting ability combined with achievement. While the coach uses first-person plural “we” to refer to their prior actions and mutual goal, she uses first-person singular “i” and matching pronouns to introduce her piece of agenda-relevant information, i.e., her wish to specify the goal, and to account for it. She claims deontic authority with her information delivery statement but allows for the possibility of client disagreement in the use of mitigating particles and conjunctive mode with the modal “would” (l.3). In the following, the client responds with an acknowledgement token when the coach continues to summarize goal-relevant information from her notes.

#### 4.1.2. Commenting on own action (*n* = 15)

15 out of the 36 examples of “Delivering Agenda Information” belong to the category “Commenting on own action.” In the majority of cases, the coaches make their actions transparent by informing clients about the fact that they are (about to be) taking notes. This transparency regarding their actions is even mirrored in their choice of vocabulary (*visualize, make visible, display*, …). Again, we find various temporal adverbs (*now, again, today*, …) in these declarative informing statements. The coaches position themselves as the agents of the action via first-person singular pronouns and active voice in all examples. Present tense indicative forms are used throughout. Often, the coaches minimize the impact of their actions on the overall activity with *a bit, some, only, just,* etc. We also frequently find hesitation markers, pauses, audible breathing as well as accounts whereby coaches might want to mitigate their explicit assumption of higher deontic stance: They often name aims (*to visualize/display/note down the concern*) or give reasons (*to check, so that I can get back to this, so that I can track our progress more easily, to structure this*, *in order to keep track,*…) stressing the positive impact of taking notes for the clients and the process as such. Indeed, while information deliveries do not present instances in which the coaches’ deontic authority—as the persons in charge of the process and of the action—can be easily challenged, the professionals still account for their doings for the benefit of their clients.

**Example 2:** Delivering Agenda Information: Commenting on own action.


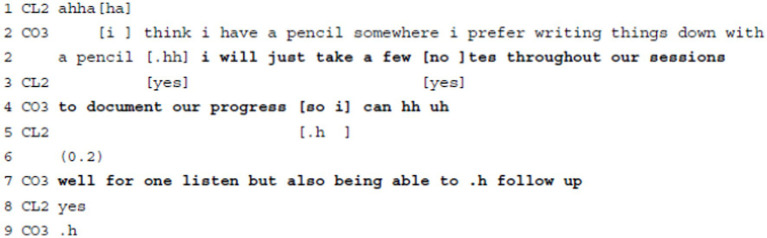


In Example 2, the coach informs the client that she will take notes. She modulates her comment in different ways, e.g., via the modal particle “just” or by reducing the extent of her action by “a few” (l.2). She stresses the benefit of her action by referring to the coaching as a mutual process (“our progress,” l.4) and by producing an account, i.e., that she does not only want to be able to listen to the client, but also to get back to aspects (“listen” and “follow up,” l.7). Using declarative statements in the present tense and indicative mode, the coach stresses her deontic authority to decide on such procedural next actions. At the same time, by referring to “our” (l.4) progress, the coach constructs her actions as beneficial for the process and, eventually, for the working alliance, too. While the coach explains her actions by drawing on her epistemic and deontic authority, she ensures transparency regarding the purpose and the addressee of these notes and thereby builds trust with the client. Besides some overlapping acknowledgement tokens (“yes,” l.3), the client produces a positive receipt of this information once the coach has finished her turn (l.8).

### 4.2. Requesting agenda information (*n* = 14)

In our corpus, agenda-relevant coaching information is not only delivered, but also requested by the coach. In their professional role, coaches have the deontic authority to ‘demand’ information in order to benefit from this knowledge. By requesting information, the coach, as beneficiary of a knowledge upgrade, seeks to gain some measure of access to the client’s (territory of) knowledge (Heritage, 2012), thus positioning the client as the agent (Couper-Kuhlen, 2014). It is on this basis—i.e., their upgraded epistemic status—that coaches can then proceed taking next procedural decisions. There are 14 such instances in the corpus.

In the context of “Requesting Agenda Information,” clients are expected to provide insights into their concern(s) and goal(s) of coaching and how they can be approached (11 examples) as well as their expectations concerning the coaches’ role and responsibility (three examples). In these agenda moves, clients are attributed a [K+] and coaches a [K-] status (with coaches’ [K-] status being lower on an epistemic gradient with requests for information than with requests for agreement, and vice versa with clients’ [K+] status (Heritage, 2012)). While coaches request information based on their deontic authority as professionals, clients’ deontic authority shows in how they react to such requests, i.e., what kind of information they offer in which form as their response.

Concerning the syntactic structure, interrogatives are found in the great majority of examples of requesting agenda information. This is in line with Heritage (2013, p. 563) who states that “(i)n contexts where an utterance formed with interrogative syntax [it] concerns information that is (primarily) within the recipient’s epistemic domain.” In terms of form, the instances display certain patterns. Requests are often phrased via modal auxiliaries and conditional *would*. We frequently find first-person pronoun *I* used by the coach as well as second-person pronoun *you* addressing the client directly.

#### 4.2.1. Defining content/goal (*n* = 11)

The category “Defining content/goal” of the coaching (session) is often (yet not exclusively) found in the first parts of the first sessions and comprises 11 examples. Along with general initiatory requests via open *wh*-questions regarding goal or concern (*What exactly is the concern? What is your goal?*), we also find more topic-specific requests for information (*What do you want to achieve with the coaching? Which of the two concerns would you prioritize? What would be useful for you?*). The clients and their wishes are directly addressed in the majority of cases, which increases response relevance even further (Stivers and Rossano, 2010), but we also find a few impersonal constructions (*But what are topics that need to be [dealt with]?*). These agenda moves showcase the client-centeredness of coaching: Knowledge about the concern lies in the clients’ epistemic domain, and hence coaches need clients’ collaboration when defining the goal. In the turns following the request proper or building on this common ground at later stages, the cooperation between coach and client in working toward clients’ goals is sometimes explicitly stressed via the use of collaborative *we* in further requests for information (*What else can we do? What would be a coaching goal that we can aim at?*) (see, e.g., Nanouri et al., 2022, p. 109).

**Example 3:** Requesting Agenda Information: Defining content/goal.


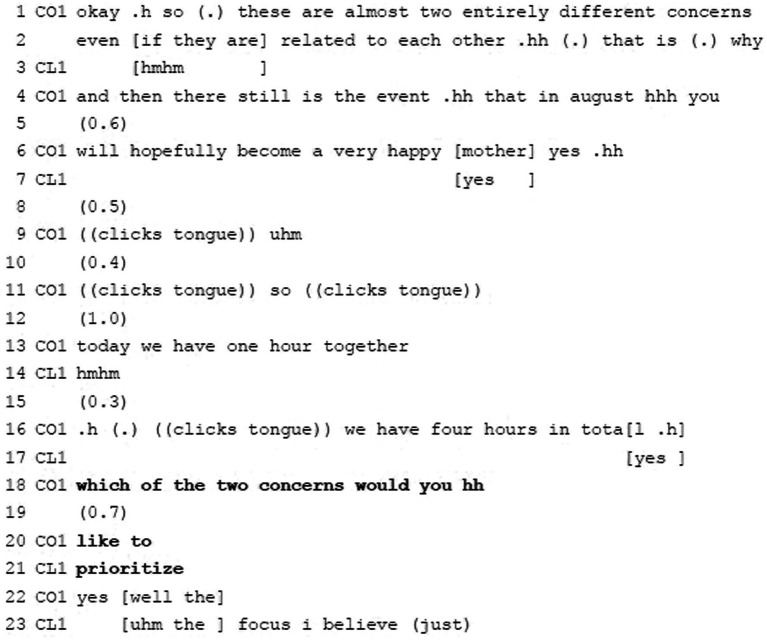


Example 3 features an agenda management move which is typical across many helping interactions (see, e.g., Boyd and Heritage (2006) for doctor-patient interaction) and is also found in coaching: via *wh*-questions, the professionals invite clients to define their concerns/goals in coaching or—as is the case here—to decide on the most important one.

Prior, the coach asked the client to elaborate on her current situation and to name coaching-relevant topics. Among others, the client explains that maintaining her focus is a major problem. The coach—ratifies the client’s elaboration with “okay” (l.1) and, based on her epistemic authority, concludes that the issues constitute two separate (though interrelated) concerns (ll.1–2). Without being prompted, the client minimally agrees with this (“hmhm,” l.3). The coach tentatively adds that the client becoming a mother soon might be another issue and finishes with a question tag seeking confirmation and thereby addressing the client’s epistemic authority (l.6). The client responds affirmatively (“yes,” l.7). Thereafter, the coach—in an information-delivery sequence—sets the time frame as regards the current session (using the temporal adverb “today” and naming “one hour” as scheduled duration, l.13) as well as the entire coaching process (referring to “four hours in total,” l.16). In each case the coach uses the personal pronoun “we” (ll.13 + 16) stressing the joint activity. The client first provides a minimal acknowledgement (l.14), and then a clearly affirmative one in an overlapping manner (l.17). Against this common knowledge regarding the time frame and the two distinct concerns, the coach, again, requests agenda-relevant information via a polite *wh*-question (leaving the client freedom of decision) so that she can continue her agenda management. She addresses the client via the personal pronoun “you” and uses conditional “would” along with the verb “like” to learn about the client’s priority. She starts off with “which of the two concerns would you hh (0.7) like to” (ll.18–20), and the client immediately provides the verb “prioritize” (l.21) to collaboratively complete the coach’s turn, which shows her attentiveness:

The joint production of an utterance, in which one speaker begins the utterance and another extends it, is a carefully orchestrated accomplishment requiring considerable attentiveness and skill from the second speaker; that is, the second speaker must be able to project when turn constructional units (…) are nearing completion and, at the same time, must be able to immediately build upon the utterance by adding an appropriate grammatical unit that semantically coheres with what has come before (…). These co-constructed utterances also have considerable social relevance, because they index a high degree of cooperation, solidarity and involvement between the participants (...). (Muntigl et al., 2013, p. 11)

The coach thus actively invites and acknowledges the client’s expert status as regards her (prioritizing the) concern. By completing the coach’s turn, the client accepts this ‘invitation’ with confidence. The coach accepts this (“yes,” l.22) and after an overlapping continuation yields her turn to the client to name the concern to be dealt with first (“the] focus,” l.23). Agreement on goals, an essential component of the working alliance, has been reached for the current session.

#### 4.2.2. Defining roles and responsibilities (*n* = 3)

Three instances of requesting agenda information explicitly relate to the role and responsibilities as a coach and (in this function) they form a subtype of “Requesting Agenda Information.” This subtype (comprising interrogatives only) explicitly addresses relational issues and is primarily bond-related.

**Example 4:** Requesting Agenda Information: Defining roles & responsibilities.


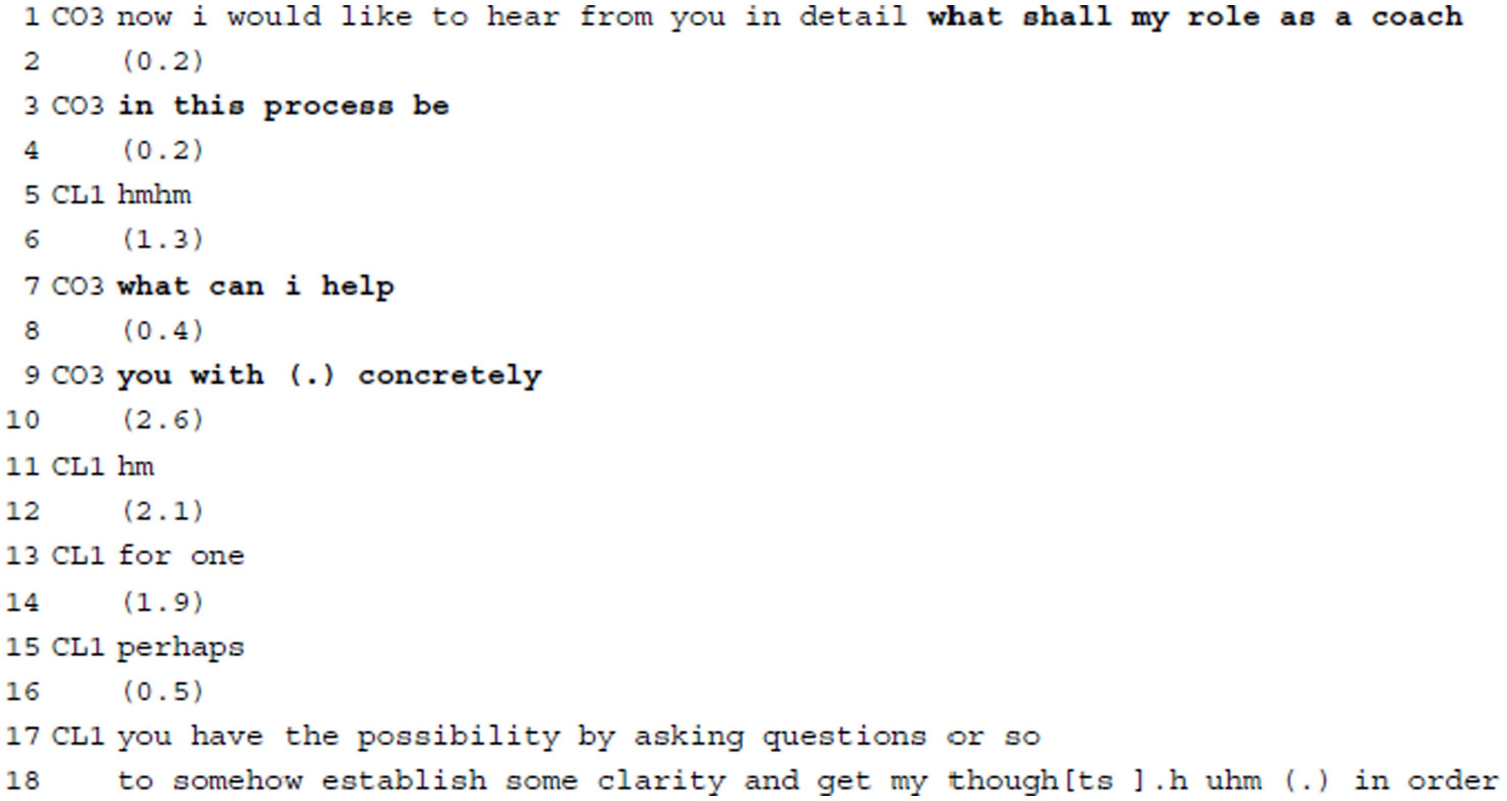


In example 4, the coach puts herself at the service of the client via using thematically open interrogatives. She thereby first requests general information about the client’s wishes regarding her role/responsibilities in the coaching process (“what shall my role as a coach (0.2) in this process be,” ll.1–3). She formulates this as an open *wh*-question and directly addresses the client, attributing both epistemic and deontic authority to her. At the same time, the coach assumes deontic authority by asking the question at this particular point in the process (“now i would like…,” l.1) and by requesting very specific information (“in detail,” l.1) thereby putting pressure on the client to provide such details. The *wh*-question is embedded in a longer, multi-turn formulation of the client’s concern (not in the excerpt) and, at first, after a 0.2 s pause, the client only provides minimal acknowledgement (“hmhm,” l.5). After another 1.3 s pause, which suggests interactional trouble (Kitzinger, 2013), the coach reformulates her question in a self-initiated self-repair and specifies her prior formulation “my role as a coach (0.2) in this process” (ll.1–3) via concretizing her role as offering help “what can i help (0.4) you with (.) concretely” (ll.7–9). While offering help accentuates that the client is in need of support and builds on the assumption that the coach can provide this help, inviting the client to specify the type of help implies that the client has an active share and responsibility in the outcome as well as sufficient knowledge regarding the kind of support needed to achieve it. This points at the traditional sharing of tasks in coaching: The coach is responsible for the process, the client is responsible for the content. That the coach intends to adjust her role (and interventions) in the coaching process to the client’s individual needs and expectations (as a form of client design, Graf and Jautz, 2022) also shows in the use of “concretely” (l.9). After a 2.6 s delay, a hesitation marker (“hm,” l.11) possibly indicating reflection and another pause of 2.1 s, the client starts to provide an answer, which covers different aspects. The structuring device “for one” (l.13) indicates a complex upcoming turn which will involve several components (see, e.g., Thompson et al., 2015, on responses to *wh*-questions). Her response shows that she is not only prepared to formulate her needs, but also has some knowledge about coaching practices, i.e., that coaches ask questions (“by asking questions,” l.17) to help clients concretize their thoughts. Her uptake is phrased tentatively with various mitigating expressions (“perhaps,” l.15; “or so,” l.17; “somehow,” l.18), which can be interpreted as an awareness of the socially challenging situation to tell a professional expert what to do. It also possibly indicates a lack of clear procedural knowledge of what the coach can actually do. At the same time, via her suggestions the client assumes some deontic authority to mold the coach’s future actions.

### 4.3. Requesting agenda agreement^3^ (*n* = 15)

We found 15 instances of coaches seeking an agreement relating to the chosen procedure. Coaches therein seek simple agreement or elicit a client’s stance in search of agreement on a suggested procedure. The frequency of this agenda move illustrates that expertise regarding the content, but also regarding the experienced adequacy of (planned or taken) measures is attributed to the clients in coaching. All of them are phrased using interrogative syntax; they are very uniform in that the coaches’ display of power does not align with their deontic authority as professional agent to, e.g., suggest a certain procedure at a particular moment in coaching. Instead, they attribute deontic authority to the clients to authorize these suggestions or reject them, i.e., procedural decisions are highly contingent on clients’ acceptance (Muntigl, 2023, p. 271). This once again showcases the client-orientation of coaching at large. The majority of the examples are either task- or bond-focused, while only few are goal-focused.

In terms of linguistic features, we find the polite use of conditional *would* (*Would this be something for you? Would this be a good moment to come to an end?*) as well as modal auxiliaries (*May I note down X? May I just briefly share what I just thought?*). The requests are mitigated (*just, a bit*) and contain temporal references indicating a short duration (*just briefly, a moment, for the time being*) and only minimal intrusion. Clients’ negative face needs are respected. Furthermore, we find some impersonal formulations (*Can it be left like that for today?*).

**Example 5:** Requesting Agenda Agreement.


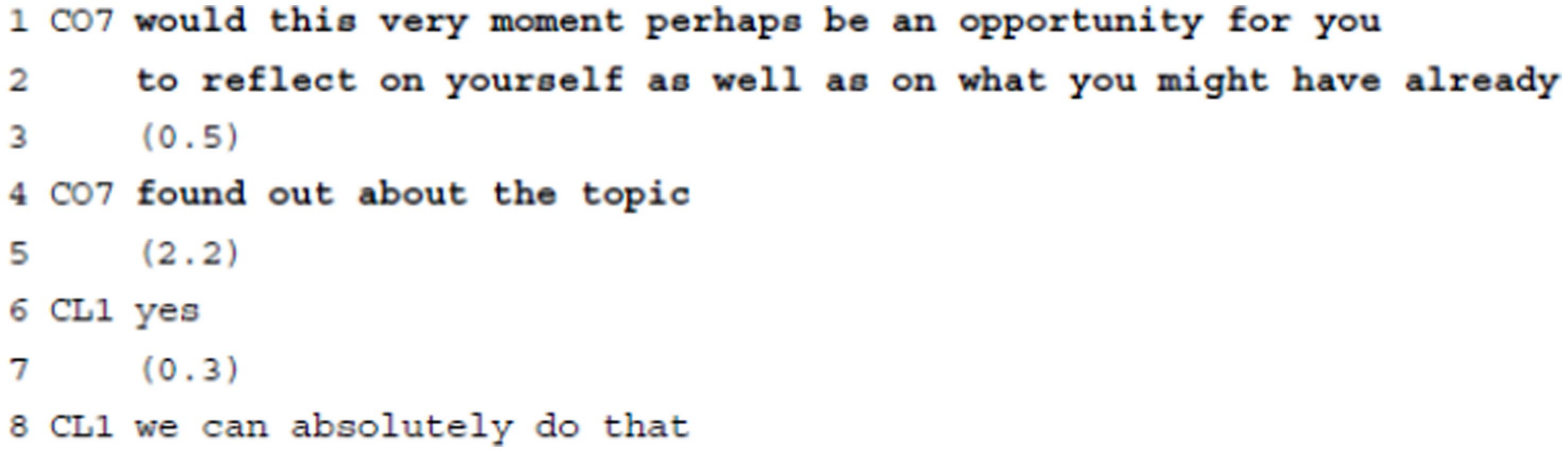


In example 5, the coach elicits the client’s stance regarding the timing of a continuation of the session (“would this very moment perhaps be an opportunity,” l.1) with reviewing what the client has already learned about herself and the topic at hand (“to reflect on yourself as well as on what you might have already (0.5) found out about the topic,” ll.1–4); he mitigates his request using a conditional form and “perhaps.” Also, he constructs the implicitly announced intervention as useful by labeling it “an opportunity.” The polar interrogative question format does not, however, question whether reviewing previous work is a useful course of action; instead, the requested agreement (i.e., confirmation, see footnote 3) only concerns whether the client considers the present moment a good time to do a review (referred to via the spatio-temporal “this very moment,” l.1). While the client is thus given the power to decide on the adequacy of the timing for the intervention (and is the agent of this decision), she is not given the power to decide on the intervention as such. Deciding on its appropriateness or adequacy remains in the coach’s epistemic and deontic domains. After a considerable pause of 2.2 s, the client first only produces the minimal agreement token “yes” (l.6), but—after another short pause—upgrades her agreement to a more enthusiastic stance “we can absolutely do that” (l.8), thereby granting the requested agreement, from which the coach benefits. Interestingly, while the coach directly addresses the client “you […] yourself” (ll.1–2), the client employs a collaborative “we” (l.8).

### 4.4. Requesting agenda action (*n* = 3)

There are three instances in our data where the coach requests agenda action rather than agenda information or agreement from the client. Requests most generally are directives with which the speaker (in our examples the coach) wants the addressee (the client) to do something: These directives “involve some future event or task to be accomplished, orient to speakers’ rights and responsibilities, and make relevant some form of acceptance or compliance by the recipient or commitment to carry out the task (…)” (Muntigl et al., 2020, p. 2). The speaker’s power to get the other person to take over some future action varies, as was argued by Stevanovic and Peräkylä (2014, p. 192):

(r)equests for action may range from orders and commands to suggestions and hints, depending most fundamentally on the extent that the first speaker may assume that the second speaker will perform the relevant action without being directly asked for it. Declarative statements do not necessarily impose any action on the recipient. Hence (…) we argue that such an interpretation is contingent on the recipient’s judgments about the speaker’s high deontic status relative to the recipient in the domain in question.

The three instances of “Requests for Action” do not contain imperatives, but two declaratives and one interrogative. In two of them, the coach asks her client to correct her if she has understood or summarized the client’s prior talk incorrectly, in the third one the coach requests the client to state her goal, thereby contributing to agenda-setting and working alliance alike.

**Example 6:** Requesting Agenda Action.


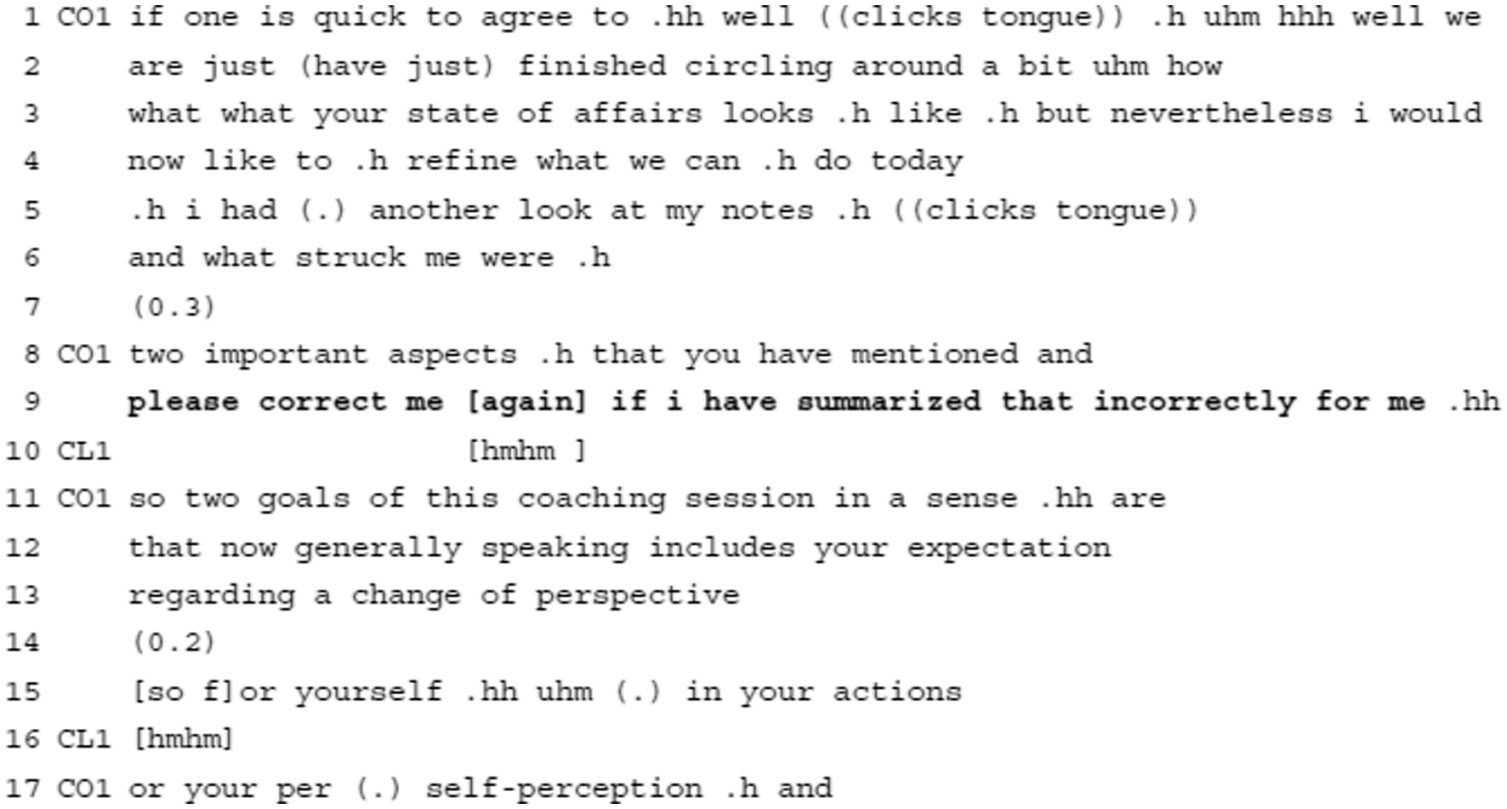


As already explained in section 4.1.1, Example 6 first features a delivering agenda information move with which the coach tries to structure the thematic focus of the session. After this preparatory move, the coach introduces an upcoming highlighting formulation (Weiste and Peräkylä, 2013) of the client’s concerns (“i had (.) another look at my notes .h ((clicks tongue)) and what struck me were .h,” ll.5–6). Before proceeding with the actual formulation (l.11ff.), though, the coach requests future agenda action from the client, asking her to correct her in case she got it wrong (“please correct me [again] if i have summarized that incorrectly for me .hh,” l.9). She thereby displays a high entitlement to request such action from the client, using the imperative mode, only slightly mitigating her directive with the adverb “please” (l.9). At the same time, she attributes both the epistemic authority to the client regarding the content of the formulation (as pertaining to the client’s epistemic domain) and the deontic authority to take agentive action (of correcting) and potentially turn the summary down. The client acknowledges the request for action in providing a minimal acknowledgement token (“hmhm,” l.10).

This request for action, i.e., for correction, clearly bears on the relationship between coach and client in the sense of both having similar rights and responsibilities. While the coach’s formulation displays an updated epistemic stance regarding the client’s concerns, she concurrently positions the client as having the epistemic authority over this domain by explicitly inviting correction. As such, the agenda move bears both on the goal-component of the working alliance given that the participants need to agree on what they should be working on as well as on the bond-component of the working alliance, i.e., on establishing a stable relationship where critique is possible. Still, the right to exert influence on the professional agent via a possible correction (i.e., an explicit other-initiated other-repair) presents a delicate interactional moment: explicit corrections are indeed dispreferred social actions that are generally avoided (Pomerantz and Heritage, 2013, p. 217). The explicit directive to do so, then, works toward minimizing the possible negative impact that a correction may have on the working alliance.

### 4.5. Suggesting agenda action (*n* = 34)

We now turn to ‘suggestions’ as another kind of directive and controlling social action. Couper-Kuhlen (2014, p. 634) distinguishes between ‘suggestions’ and ‘requests’ in that the social action ‘suggestion’ features the recipient (i.e., the client) as both the agent and beneficiary of the suggested future action, whereas the beneficiary of the action ‘request’ is the speaker (i.e., the coach) and the recipient is the agent. We found 34 instances in our corpus matching the former description. The examples are located on a continuum ranging from suggesting procedure-oriented actions to suggesting concern-oriented actions. The former refers to actions which, e.g., address the next step that needs to be completed by a certain time. Thus, their procedural relevance is propositionally highlighted, and the examples often feature temporal adverbials referring to either a specific moment (e.g., *now, at this point [in time] in the next session*) or a period of time (e.g., *briefly, until we meet again, during the next session*) which is usually used to argue for the feasibility of the suggested action. With concern-oriented suggestions, clients are invited to reflect on their goal, aspects of their personality, strengths and weaknesses, or on what has been discussed so far. Instances of “Suggesting Agenda Action” build on mutually upgraded knowledge as regards clients’ concerns/goals, i.e., are found during later stages of first sessions. The negotiation of these suggestions promotes a possible agreement between coach and client on the goal(s) and tasks of the coaching.

The coaches mostly phrase their suggestions as declaratives in the form of *you can do X* or, somewhat more directive, *I would ask you to do X*. We also find no-agent constructions in the passive voice (*a look would have to be taken at X*). In a few cases, the coach prefaces the suggestion with an explicit attribution of deontic authority to the client by emphasizing volition (*if you feel like (doing X), if you like (we can do X)*). In addition to examples showcasing the verb *suggest* (*I would like to suggest X*) or the noun *suggestion* (*my suggestion would be X*), we find verbs expressing intention, willingness or wish, often featuring conditional *would* (*perhaps you would like to do X*). We also find some examples in which the coaches, in a pre-sequence (Schegloff, 2007), ask for the clients’ permission (*if you permit*) or their agreement (*if you want to*) before uttering the actual suggestion. Yet other examples are more straightforward and emerge locally without any preparatory moves. Following the actual suggestions, we find various cases of accounting, where coaches stress the benefits for the clients and their goals in coaching (e.g., *maybe this way it becomes more transparent for you, perhaps first steps can be derived from this, perhaps it is also helpful for you to set milestones*).

While the agent and the beneficiary of the suggested future coaching action is always the client, as expressed in the use of the second-person singular pronouns (*you decide, you can ask people, your task would be to do X*), we also find suggestions that draw on first-person plural pronouns (*we could consider X, we can take a closer look at X, we ask X*). The coaches’ suggestions often feature mitigating devices (*perhaps, a bit, just*) and hesitation markers, pauses and breathing, which render their turns rather tentative in nature. As such, coaches—while having the relevant (knowledge and) power as professional experts of appropriate next steps– mostly do not publicly display a stance congruent with their (epistemic and) deontic status as professionals (Stevanovic and Peräkylä, 2014, p. 189).

**Example 7:** Suggesting Agenda Action.


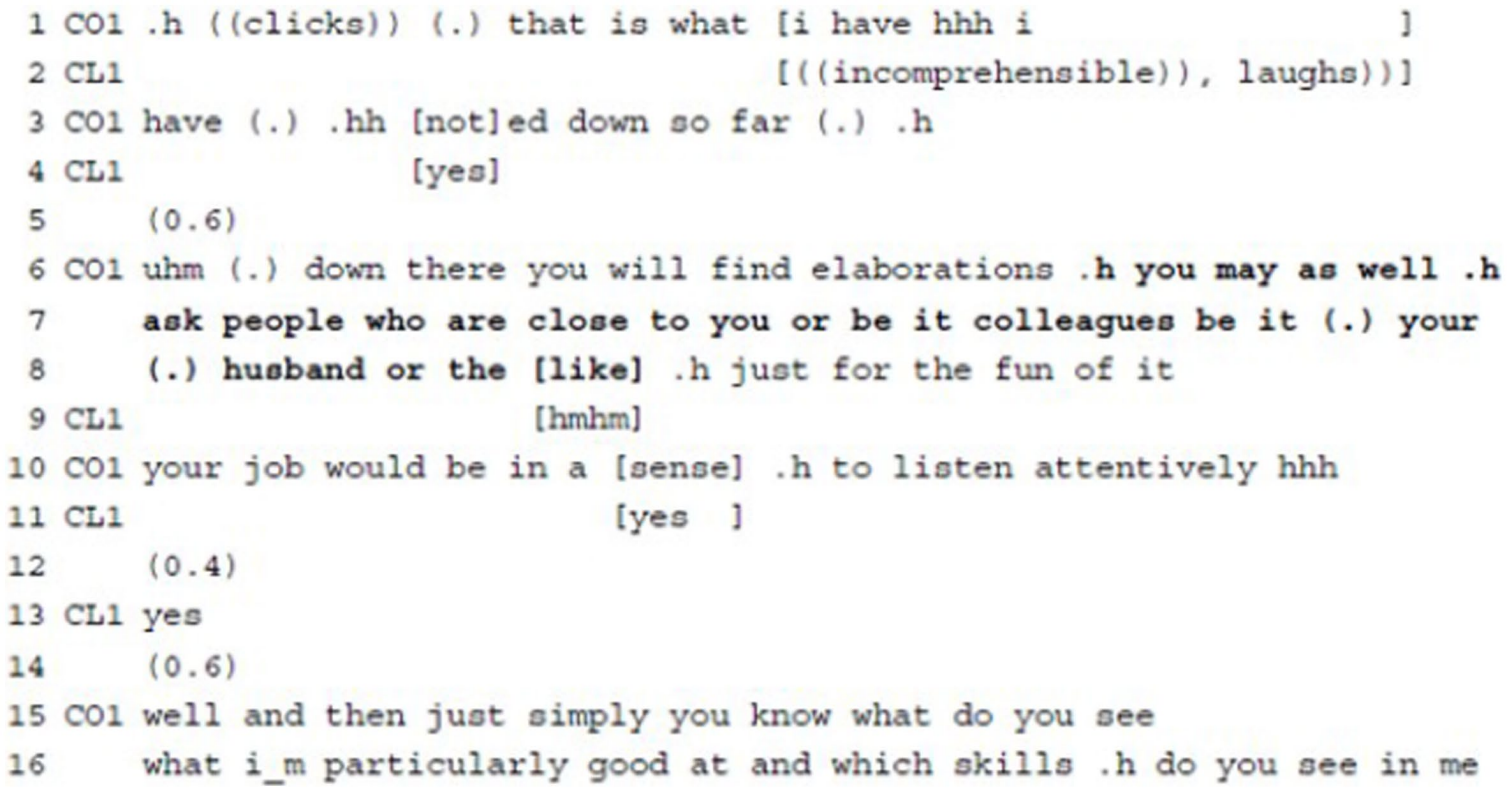


Example 7 is an instance where the coach suggests a concern-oriented action for her client. About 5 min before the end of the session, the coach summarizes her notes on what the client has said and then continues with some homework for the client. After referring to some explanation entailed in a document for the client, the coach suggests the first version of a task, i.e., that the client asks family and friends what they consider to be her strengths, and next, she specifies the client’s task (“your job would be,” l.10) as to listen to what they say. The first part of the task is phrased as a possibility for a client action (“you”) involving the modal verb “may” (l.6) and comprises several alternatives as to whom the client might ask, leaving it open for the client to decide exactly who would be most appropriate for the exercise (“people who are close to you or be it colleagues be it (.) your husband or the [like],” ll.7–8). The coach minimizes the costs of the suggested task for the client (Couper-Kuhlen, 2014, p. 626) and highlights the easiness in proceeding by strongly mitigating her utterance with the adverbials “just” and “for the fun of it” (l.8). In this same minimizing sense, she emphasizes that the client’s actual task would be to “in a [sense] .h to listen attentively hhh” (l.10) to her friends and family. In spite of this, the coach leaves it up to the client to decide whether this ‘easy homework’ will be completed or not: she designs her utterance using the conditional, thus stressing the optional nature of the suggested task. The client reacts with overlapping acknowledgement tokens (ll.9 + 11) and a positive polar interjection (“yes,” l.13). The coach then precises how the client’s asking could be “simply” done, providing candidate questions as explanations (l.15ff.). She “takes on the client’s voice thus speaking as if she were paraphrasing or quoting the client’s message” (Muntigl, 2013, p. 7 on therapy), using direct speech and the first person singular, and details what the client could say to the people in question. This creates both immediacy and emotional involvement and adds transparency to her task.

### 4.6. Offering agenda action (*n* = 9)

Another nine instances of agenda management were classified as “Offering Agenda Action.” Just as with suggestions (chapter 4.4), the client is the one who benefits from the named action; yet, unlike with suggestions or requests, the coach is the agent of the offer. This makes offers commissive actions: The coaches commit themselves to carrying out the future action in question, which refers to “the transfer of an object or a service” (Couper-Kuhlen, 2014, p. 249). Future actions often address some kind of trouble or problem that emerges locally or has previously been made explicit (Couper-Kuhlen, 2014, p. 634); another type refers to offers with respect to troubles or, more generally, topics that emerge alongside the interaction (without the original intention of making an offer; Drew, 2013, pp. 6f.). Both formats are found in the current data: in two cases, the offers address a locally emerging issue and include the client in the future action (e.g., they can ask questions). The other offers relate to possibilities of outsourcing certain matters or tasks instead of spending coaching time on them (e.g., coaches offer to send the clients background information) or to material, exercises or activities to be integrated into the session at hand. The preferred way of responding to an offer is accepting it (Couper-Kuhlen, 2014, p. 624), and indeed, the clients respond with explicit positive uptakes in all cases (*yes, yes-yes, okay*). Regarding their contribution to establishing the working alliance between coach and client, all instances of this social action type are either task- or bond-focused.

Offers, especially locally emerging ones, often take the form of a declarative (Drew, 2013, pp. 6f.). This is the case with all nine examples in our corpus. Apart from one impersonal example, all offers comprise first-person singular pronouns *I* for the coach as the (future) agent. And all but one example feature characteristics of mitigation in the sense of pauses or audible breathing, conditional mode, auxiliaries *would, may* or adverbials such as *a few*, *a bit*, *perhaps*. While offers are preferred actions compared to requests (Levinson, 2013, p. 115), coaches still—despite offering something beneficiary for the clients—thus display a deontic stance incongruent with their deontic authority here.

**Example 8:** Offering Agenda Action.


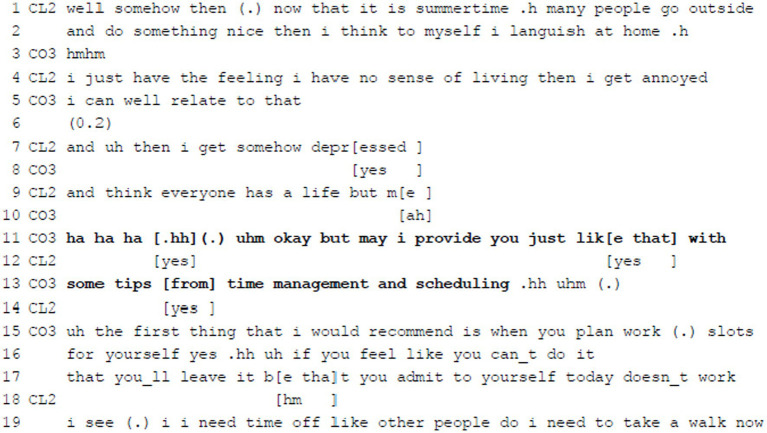


In Example 8 the client explains that she has problems with her work-life balance (ll.1–9). The coach affiliates with the client and voices understanding for her situation (“i can well relate to that,” l.5). The client adds that she feels depressed and like not having a life of her own (ll.7 + 9). In reaction to this, the coach offers to take action, i.e., to give some tips regarding time management (ll.11 + 13). Before the actual offer, the coach starts her turn with affiliative laughter, then breathes in, pauses and hesitates, and uses the acknowledgement token “okay” and adversative “but” (l.11) to introduce a measure against such feelings expressed by the client. Giving tips implies expertise and epistemic authority and underlines an asymmetric and hierarchical relationship. However, the coach downplays her authority and orients toward the client by asking for permission (using the modal verb “may,” l.11), mitigating her offer (“just,” l.11; “some,” l.13) and by using hesitation markers. Only thereafter the coach starts naming her tips. There is, however, no pause which would give the client the chance to grant permission. However, since the client has provided agreement in overlap, the coach can build on this positive uptake and elaborates different recommendations such as to reserve slots for herself in her work schedule and to accept that there are days when it is not possible to stick to a scheduled plan.

### 4.7. Proposing agenda action (*n* = 6)

Our corpus features six examples which were classified as proposals, i.e., as “Proposing Agenda Action.” These instances are characterized by coach and client both being agents and beneficiaries of the proposed future action, which is documented in the use of the first-person plural pronoun *we* used throughout this category. Via the use of conditional and modal verbs (*we can, could*), the proposals are all framed as options, respecting the negative face of coach and client alike. Moreover, they are all metapragmatic statements of proposed future actions in that these instances realize the ‘discourse on coaching,’ not the ‘coaching discourse’ (Graf, 2019, p. 290). This is reflected in the verbs from the semantic field of communication, e.g., *coordinate*, *sort out*, *agree on* or *tackle a topic.* In their structuring function, these agenda moves also often contain temporal options for coach and client such as *next session* or *second step*. In these proposals (and their uptakes, ranging from minimal acknowledgment to *yes, love to*) coaches frame possible next agenda steps as open for discussion and explicitly involve the client in the decision (e.g., *if you want to*), thereby mitigating a potential face threat and enhancing the clients’ freedom of action. While proposing next agenda steps bears on the task-component of the working alliance, the fact that these moves create transparency and commitment on the side of the client promotes the bond between the interactants.

**Example 9:** Proposing Agenda Action.


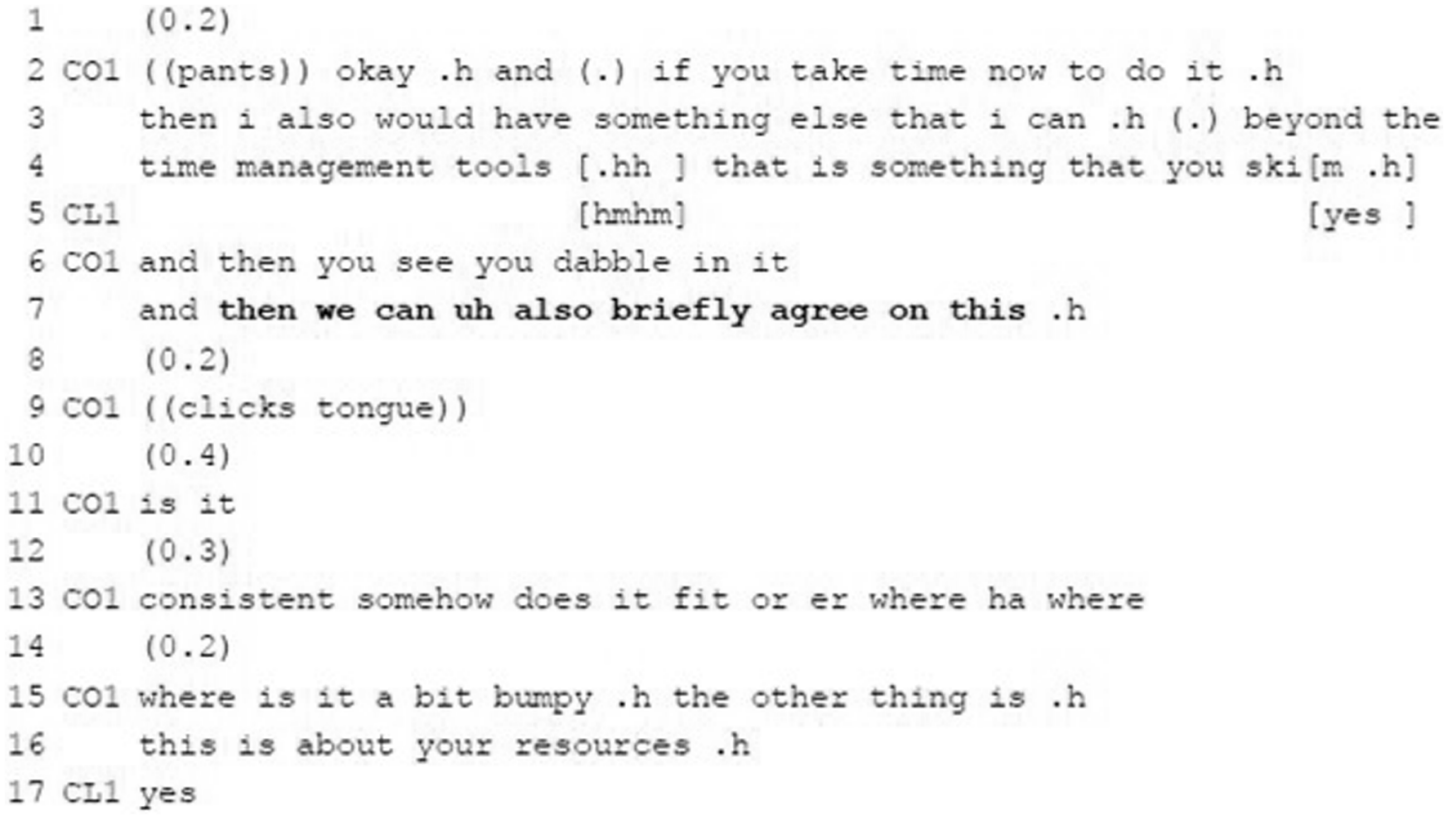


In Example 9 the coach offers some additional material on time management tools which the client can read and try out. This serves as a basis for agreeing on whether the tools fit for the client. The proposal (l.7) contains the temporal adverb “then,” referring to the future action. While making such a proposal documents the coach’s deontic authority, integrating the client in the agenda management by phrasing the proposal as possibility (via the modal “can,” l.7), by employing the first-person plural pronoun “we” and the verb “agree […] on” (l.7) stresses agenda management as joint activity in which client’s deontic authority is upgraded. The coach also mitigates the effort or time investment by adding “briefly” (l.7), which will make the proposal more easily acceptable for the client. The client provides continuers (“hmhm,” “yes,” l.5) during the coach’s turn though prior to the proposal. However, she does not immediately provide a positive uptake regarding the proposal itself. After some pausing, the coach offers further explanations what ‘agreeing on’ means (ll.13–16) emphasizing that the proposed has to fit the client. Thereafter, the client agrees (“yes,” l.17).

## 5. Discussion

The interactional trajectory of agenda-setting in first sessions of dyadic business coaching is closely linked to the domains of knowledge and power of the participants and how these bear on the participants’, i.e., on coaches’ and clients’ momentary relationship (Stevanovic and Peräkylä, 2014, p. 187), e.g., on the mutual updating of epistemic statuses. In this vein, the seven social actions presented here (roughly) replicate a stepwise, ‘natural’ order of agenda management: The participants first need to reach a common understanding of the content, the procedure and their relationship before they can negotiate taking (future) coaching actions: coaches as professional experts must inform clients on what to expect from coaching as a helping format and, in turn, they need information from clients why they came to coaching and what they expect of them as professionals. This mutual upgrading of coaching-relevant knowledge prepares the ground for next steps in coaching: it adds to the deontic authority of coaches to impose future actions on the client via, e.g., suggesting a certain intervention as part of working on the tasks of coaching; it also adds to clients’ deontic authority to take informed, or “consensus-based” decisions regarding these suggested interventions (Frankel et al., 2013; Muntigl et al., 2020). It was beyond the scope of this paper to analyze the positioning and sequencing of the different social actions in detail and providing statistical evidence, yet this order of agenda actions proves a pattern (albeit not a strict order) recurring at different stages in the different first sessions.

The distribution of epistemic authority concerning procedure and content showcases the ‘division of labor’ as claimed in coaching practice literature (e.g., Barczynski, 2018, p. 9), viz. how coaches shape coaching-relevant knowledge, entitlements and orientations to knowledge and knowledgeability and overall render coaching a client-centered interaction. Yet, in line with Vehviläinen (2003) and Vehviläinen and Souto’s (2022) observations for counseling and Nanouri et al’s. (2022) observations for adult education and therapy trainings, the professional coaches display a ‘double orientation,’ i.e., they orient to being collaborative, while retaining their authority. Though set within the larger socio-cultural framework of democratizing expertise and client participation and, more coaching-specifically, an ideology of help for self-help and dialogue at eye level (e.g., Jautz, 2017), coaches exercise a legitimate degree of power due to their epistemic and deontic status as professional coaches, and clients endorse such expertise and power. While strategies of face-saving and politeness thereby play a role for both participants, future research must zoom in on these aspects of relational management.

As such, the social actions found in the data promote and underlie the working alliance in coaching and do not only ‘do’ agenda-setting, but they also ‘do’ working alliance: Agreeing on the goal(s) of the coaching (e.g., requesting information from clients regarding their concerns), assigning task(s) to reach these goals (e.g., suggesting agenda action via certain interventions), and establishing a bond between coach and client (e.g., delivering agenda information regarding the structural set-up of coaching).

### 5.1. Delivering agenda information

In more detail, in “Delivering Agenda Information” (somewhat less than one third of all social actions; *n* = 36), coaches inform clients about possible content of their coaching interaction or about the temporal framing of the session or the process. Moreover, coaches also inform clients what they themselves are/will be doing in the sessions. As outlined by Graf (2019, pp. 75ff.) in the context of the basic activity “Defining the Situation” and more specifically, in the context of the communicative tasks “Methodological and Procedural Framing of Coaching” (Graf, 2019, p. 85) and “Temporal Framing of Coaching” (Graf, 2019, pp. 115ff.), such informings are very often done explicitly via meta-pragmatic framing practices. What was outlined by Peräkylä (1995, p. 98, emphasis in original) for counseling similarly holds true for coaching: “(w)e—as ordinary members of Western societies—do not know what happens in counseling with the same precision as we know what is going on in a doctor’s surgery or in a lecture hall. For the clients, then, what the general goals of a counseling session are may be more or less *opaque*.” Thus, “Structuring content/session/process/coaching” (*n* = 21) builds on coaches’ epistemic authority as professional coaching experts and enables an upgrade of clients’ epistemic status with respect to how coaching will proceed, i.e., it reduces the opaqueness of coaching by creating a thematic and procedural common ground. In “Commenting on own action” (*n* = 15), coaches inform clients about the rationale of a certain action, primarily of them taking notes during the session. This meta-pragmatic framing strategy creates transparency for the clients and pays tribute to their entitlement to know the motivation for coaches’ actions. Moreover, in sight of the triadic constellation of (most) coachings (Graf and Jautz, 2019), taking notes could be experienced as breeching the confidentiality between coach and client; informing clients about the ‘addressee’ thus helps to build trust. While delivering coaching-relevant agenda information prepares the ground for agreeing on goals and tasks in coaching, it also enables establishing a trustful bond between coach and client. Particularly the latter aspect of delivering agenda information seems highly relevant in the context of the still unresolved professional status of coaching and the resulting insecurity for clients about what to expect. The fact that most instances of agenda-setting in our data (*n* = 36) belong to the social category of “Delivering Agenda Information” can be interpreted as a form of client-centeredness in its reading of democratizing the professional—client relationship.

### 5.2. Requesting agenda information

We found 14 instances of “Requesting Agenda Information” to implement agenda-setting in coaching. In this category, clients with their subjective life experiences are ascribed a [K+] status in the dyad and thus are requested to upgrade coaches’ epistemic status with respect to why they came to coaching, what goals they want to pursue with coaching (“Defining content and goals,” *n* = 11) and also what coaches should specifically be doing for them (“Defining roles and responsibilities,” *n* = 3). Such concern and goal elicitation via, e.g., *wh*-questions represents a core agenda move discussed in existing literature on other helping formats, too (chapter 2.1) and represents, following Silverman’s argument (Silverman, 1997, p. 93) “(…) a normatively encouraged strategy of client-centeredness (expressed in allowing the patient to nominate the agenda.” In addition, the personalization of services for clients showcases the concept of ‘client-design’ in coaching (Graf and Jautz, 2022). Concurrently, it attributes a high level of self-reflexivity to the clients, who, in addition to elaborating on their concerns and goals, are considered knowledgeable enough to specify coaches’ contributions to achieving their goals.

### 5.3. Requesting agenda agreement

In terms of frequency, “Requesting Agenda Agreement” is even slightly more common than “Requesting Agenda Information” (*n* = 15). With this social action, coaches seek agreement from clients with respect to the (temporal, structural or emotional) adequacy of taking next procedural steps or actions suggested by the coaches. Unlike for the other subtypes, where clients enter coaching with a pre-existing relative epistemic advantage, clients’ [K+] status here is contingent upon their upgraded epistemic status with respect to the locally ensuing interaction with the coaches. Although coaches here attribute the rights, responsibilities and also the obligations to know to the clients, the procedure to be evaluated is introduced by them in the first place on the basis of their professional epistemic and deontic authority. Still, clients are authorized to influence and participate in decisions regarding the thematic and interactional trajectories of the coaching encounter as a form of client-centeredness. Agreement on goals and tasks as essential components of the working alliance require eliciting the ‘reason for visit’ in the first place alongside the negotiation of adequate steps.

Agenda-setting is not only an information- and agreement-oriented joint activity for establishing a trustful bond (predominantly via creating transparency for clients) and successfully working on clients’ goals (predominantly via mutually upgrading the participants’ epistemic statuses), but also includes (first) intervening steps to work on what has been agreed on. Action-oriented agenda moves bridge the gap between the definitional phases (i.e., the basic activities “Defining the Situation” and “Building the Relationship”; Graf, 2019) and the actual coaching work on the concern, i.e., the basic activity “Co-Constructing Change”; Graf, 2019). 52 of the 117 examples serve this purpose by requesting, suggesting, offering or proposing (future) agenda action.

### 5.4. Requesting agenda action

“Requesting Agenda Action” is rare in the data. In two of the three instances coaches ask clients to correct them in case their summaries are not sufficiently anchored in clients’ original concern or goal elaborations. These agenda moves represent the most explicit options for clients to display their epistemic dominance and authority regarding their concern. While coaches in these instances are the ‘beneficiaries’ of the possible future action ‘correction,’ it is the clients who will ultimately benefit from possible adjustments to the agenda if the feedback requested is incorporated; requests as dispreferred directive actions are thus made acceptable. Clients as co-experts are granted—as a form of dialogue at eye level—both epistemic and deontic authority in these instances and are empowered to actively correct the thematic agenda as suggested by the coach. This is in line with more recent trends of client empowerment and ‘flat’ hierarchies (Nanouri et al., 2022, p. 96). Yet, the request to do so still comes from the coach, and the clients’ responses in our data point to the socially challenging situation to flip the responsibilities: “(h)owever, power does not simply vanish from our working contexts and although the hierarchy between trainers and trainees [or coaches and clients, SJ et al.] can be softened it cannot vanish” (Nanouri et al., 2022, p. 110). Still, an explicit invitation to correct a professional expert bears on the relationship between the participants and further showcases the client-centeredness and division of labor in coaching.

### 5.5. Suggesting agenda action

The next coach-initiated social action that helps set and manage the coaching agenda is “Suggesting Agenda Action.” With 34 examples this social action represents the second most frequent agenda move in coaching: once clients’ concerns/goals have been agreed on, coaches make suggestions how to continue with the coaching procedure and/or how clients’ concerns can be worked on to achieve transformation and change. Suggesting agenda actions thereby implements the task component of the working alliance. In doing so, coaches draw on their upgraded epistemic status as regards clients’ individual concern(s) and more generally on their professional stock of knowledge. Suggestions resemble requests in that they are directive speech acts and in that the addressees (the clients) are the agents of the future action, yet, with suggestions, the clients are also the beneficiaries of that action (e.g., by reflecting on their skills as a possible next step in coaching). Due to this difference, suggestions are less dispreferred than requests: Clients ‘work on their own account’ rather than for the coaches’ benefit. Given that clients enter coaching and the asymmetrical and hierarchical relationship with their coaches with a willingness to change (Whitworth et al., 1998, p. xix), one might expect clients to act as suggested by the professional authority. Nevertheless, suggestions are often prepared or accounted for via reference to an upgraded shared coaching-relevant knowledge or via explicating possible benefits for the clients and their concerns. This interactional trajectory renders the suggestion less likely to be refused. What is more, even though coaches claim the deontic authority to influence the further development of the coaching process, the turn design of their suggestions often downgrades their deontic status (suggestions are delivered tentatively in the conjunctive mode, and designed with high contingency, Muntigl et al., 2020). Concurrently, the turn design upgrades clients’ deontic authority, also including them in the decision process on procedural or concern-oriented next steps via, e.g., the use of inclusive *we*. Across the data, suggestions are thus realized predominantly via collaborative, power-sharing practices that advance clients’ autonomy and centeredness in co-designing their change process (see Nanouri et al., 2022, p. 96 for adult learning).

### 5.6. Offering agenda action

Besides suggesting agenda actions, coaches also offer agenda actions. In the nine instances of “Offering Agenda Action,” coaches—via commissive speech acts—put themselves (as agents marked by the use of the first-person singular pronoun *I*) at the service of their clients, who will benefit from actions such as sending material or giving tips. While offers generally exist in three formats (Levinson, 2013, pp. 115f.; Couper-Kuhlen, 2014, p. 634), in our data only two emerge over the course of talk as possible “additional services” in the context of issues that have been worked on together and where coaches, mostly toward the end of these negotiations, tentatively offer some extra information. Services that go beyond the proper coaching format, such as providing additional material are outsourced and go beyond the control of the coach. Coaches trust their clients to make good use of this opportunity, which, in turn, means that they consider clients on an equal footing with them. Such offers thus empower clients and implement client-centeredness. Moreover, offering agenda action also emerges more locally with respect to troubles or, more generally, topics that surface during the interaction (without the original intention of making an offer) (Drew, 2013, pp. 6f.). Clients are offered the possibility to ask questions, or coaches offer to summarize important aspects. Future actions by coaches offered to help ‘improve’ the concrete interaction with their clients can be interpreted as affiliative actions bearing positively on the working alliance, particularly on further establishing the bond between the participants. The preferred response to an offer is acceptance, and this is granted by clients in (upgraded) positive reactions in all present examples.

### 5.7. Proposing agenda action

And, finally, agenda-setting in coaching is also implemented via the social action “Proposing Agenda Action,” of which we find six instances in our data. These agenda moves best illustrate agenda management as a joint activity as coaches and clients are not only both agents of the proposed (future) agenda action, but they also both benefit from it. The proposed actions in our corpus all relate to organizational issues regarding the ensuing coaching work on clients’ concerns and add to the transparency of what coach and client can do together and when or how they can do it. While transparency—against the background of the overall opaqueness of coaching (Peräkylä, 1995 for counseling and Graf, 2019 for coaching)—helps to strengthen the trustful bond, it also underlies reaching an agreement on the tasks to carry out next. And although these proposals are always made by the coaches, which exemplifies their professional power to introduce possible future actions, they are all framed as possibilities in the conditional form with the inclusive *we* indicating a sharing of deontic authority as regards the next agenda steps. The clients acknowledge this and provide affirmative uptakes and thus contribute to taking agenda management one step further.

## 6. Conclusion

The analysis of five first sessions of business coaching offered first insights into interactional agenda management as joint activity by coaches and clients. Agenda-setting emerged as a frequent, far-reaching, complex and instrumental activity in coaching. For the current paper, we focused on coaches’ initiating actions and found 117 instances of their agenda-setting across the data, which were classified into seven superordinate social actions plus subtypes. The most widely investigated social action across CA-based research on agenda-setting are requests for information sequences (see also Fleischhacker et al., in prep). Yet, the data evinced six additional pertinent agenda managing actions beyond collecting and prioritizing relevant concerns during the problem presentation phase of encounters. What is more, we were not only interested in how coaches employed collaborative and client-centered coaching-specific agenda-setting and management. We were also concerned about how these practices contributed to ‘doing’ the working alliance in coaching, i.e., which component of the working alliance they interactively co-construct and to what extent clients participated in planning and developing content, procedure and relationship. A case in point were the frequent instances of delivering agenda information or metapragmatic framing strategies on structure, content and procedure that promoted transparency for clients and thus helped create a trustful bond. Bordin (1979, p. 252) argued that “(…) the working alliance between the person who seeks change and the one who offers to be a change agent is one of the keys, if not the key, to the change process (…).” More detailed research into the sequentially structured practices that underlie agenda-setting and promote the working alliance (e.g., how coaches prepare for agenda-setting or respond to clients in third position) would then offer valuable insights into how the change process in coaching transpires within and across individual sessions. Combining micro-level interaction insights and the effects of agenda-setting and the working alliance on coaching outcomes and client satisfaction would, however, be a promising path that only an interdisciplinary team of linguists and psychologists could embark on.

The breadth of the current research focus comes at the cost of the amount, the depth and detailedness of the analysis. More micro-level analysis of the individual social actions and their sequentially structured practices is necessary with respect to a close analysis of how agenda-setting is prepared in the turns leading up to the agenda move (as target action) and how it is further processed by the participants in second and third positions (Peräkylä, 2019). With 117 instances, our sample of agenda-setting practices was relatively large. This attests to the importance of agenda-setting in coaching both against the background of its still unresolved professional status and the resulting insecurity for clients with respect to structure, set-up, etc. and against its client-centered orientation that clients participate in planning, developing and structuring not only content, but also procedure. The latter shows in clients’ own agenda management. When examining the first sessions for agenda management actions, we found 10 instances by clients along with the 117 instances by coaches. Analyzing clients’ contributions to agenda management and the interplay of coaches’ and clients’ actions will be of special interest to provide a complete picture (Graf et al., in prep). Moreover, carving out how face needs inform the participants’ epistemic and deontic stances when managing the agenda deserves empirical attention. Furthermore, we only included five first sessions. Extending the data set beyond first sessions to entire coaching processes represents a necessary next step, too.

## Data availability statement

The original contributions presented in the study are included in the article/Supplementary Material, further inquiries can be directed to the corresponding author.

## Ethics statement

Written informed consent was obtained from the individual(s) for the publication of any potentially identifiable images or data included in this article.

## Author contributions

SJ and E-MG examined the corpus for the occurrence of agenda management by coaches and clients and assigned them to the social actions. The categorization was critically discussed with MF and FD, who also substantially contributed to the detailed analysis of the chosen examples and the discussion and interpretation of the findings emerging from the categorization. E-MG was mainly responsible for the theoretical background and the discussion, while SJ was mainly responsible for the analyses. MF and FD took special care of examples and references. SJ produced the final edited manuscript. All authors contributed to the article and approved the submitted version.

## Footnotes

^1^We use ‘agenda-setting’ in a broad sense, encompassing differentiations such as ‘agenda eliciting’ and ‘agenda reframing’ (Hood-Medland et al., 2021) or ‘agenda mapping’ and ‘agenda navigation’ (Gobat et al., 2015); concurrently, we use ‘agenda-setting’ and ‘agenda management’ synonymously.

^2^*Questioning Sequences in Coaching* (I 4990-G) is funded by the Austrian Science Fund (FWF), the German Research Foundation (DFG) and the Swiss National Research Foundation (SNF) and runs between 2021 and 2024. The project aims to shed light on the nature of questioning sequences in business coaching as well as their change-inducing potential, combining linguistic and psychological perspectives and using mixed methods to do so. More information regarding corpus and project can be found on the official project website: https://questions-in-coaching.aau.at/en/.

^3^We use ‘agreement’ as an umbrella term here which corresponds to several sequence types (such as requests for confirmation or requests for information with stance elicitation); their overarching function, however, is to seek for agenda agreement.
