# Supplementary material for: Agenda-setting in first sessions of business coaching—a focus on coaches’ practices to manage the agenda and establish the working alliance
Source: Front Psychol. 2023 Oct 9;14:1232090. doi: 10.3389/fpsyg.2023.1232090 (PMC10590917; doi:10.3389/fpsyg.2023.1232090)
Supplement: Supplementary file 1 [file Data_Sheet_1.pdf]

Agenda-setting in first sessions of business coaching – A focus on coaches' practices to manage the agenda and establish the working alliance

Sabine Jautz[1*], Eva-Maria Graf[2], Melanie Fleischhacker[2], Frédérick Dionne[2]

[1]Department of English, Faculty of Arts and Humanities, University of Siegen, Siegen, Germany

[2]Department of English, Faculty of Humanities and Education, University of Klagenfurt, Klagenfurt, Austria

***Correspondence:**
Sabine Jautz
sabine.jautz@uni-siegen.de

Original German Data

Example 1: Delivering Agenda Information: Structuring content/ session/ process/ coaching

CO1 wenn ma schnell bereit is .hh gut ((schnalzt)) .h ähm hhh ja jetzt äh
si äh sim ma (ham ma) noch mal so_n bisschen umkreist äh wo
wie wie so ihre sachlage .h is **.h etz würd ich aber doch ganz gerne**
noch mal .h ein bisschen präzisieren was wir heute .h machen können
.h ich hab mir noch mal (.) meine notizen durchgeschaut .h ((schnalzt))
und da kamen mir so .h
(0.3)
CO1 zwei wichtige punkte .hh was sie gsagt ham und da korrigieren sie mich
bitte auch [wieder] wenn ich mir des falsch zusammengefasst hab .hh
KL1 [hmhm]
CO1 also so zwei anli liegen gewissermaßen in diesem coaching .hh
dass sie also mal ganz generell sie erwarten über_s coaching
mal en perspektivenwechsel

Example 2: Delivering Agenda Information: Commenting on own action

1 KL2 ahha[ha]
2 CO3   [ich]glaube ich hab noch n bleistift ich schreib lieber mit bleistift
2   [.hh] **ich mach mir einfach so für unseren verlauf n paar [not]izen**
3 KL2 [ja]   [ja]
4 CO3 **[damit i]ch ihnen hh äh**
5 KL2 [.h   ]
6   (0.2)
7 CO3 **zum einen auch zuhören kann aber auch** .h **nachhaken kann**
8 KL2 ja
9 CO3 .h

Example 3: Requesting Agenda Information: Defining content/ goal

1 CO1 okay .h also (.)des sind fast zwei verschiedene anliegen
2 au [wenn die was] miteinander zu tun haben so .hh (.) des(.)halb
3 KL1 [hmhm]
4 CO1 und dann gibt_s ja noch den hintergrund .hh dass im august hhh sie
5 (0.6)
6 CO1 hoffentlich sehr glückliche [mutter] werden ja .hh
7 KL1 [ja]
8 (0.5)
9 CO1 ((schnalzt)) ähm
10 (0.4)
11 CO1 ((schnalzt)) also ((schmatzt))
12 (1.0)
13 CO1 ((schnalzt)) also ((schmatzt))
14 KL1 hmhm
15 (0.3)
16 CO1 .h (.)((schnalzt)) insgesamt ham ma vier stunden zei[t .h]
17 KL1 [ja]
18 CO1 **welches dieser beiden anliegen würden sie denn hh**
19 (0.7)
20 CO1 **gerne**
21 KL1 **priorisieren**
22 CO1 ja [also des]
23 KL1 [äh der] fokus glaub i (eben)

Example 4: Requesting Agenda Information: Defining roles & responsibilities

CO3 jetzt möchte ich (.) von ihnen auch noch ganz genau hören was
was sie sich wünschen **was soll meine rolle als coach sein**
(0.2)
CO3 **in diesem prozess**
(0.2)
KL1 hmhm
(1.3)
CO3 **wobei**
(0.4)
CO3 **kann ich ihnen konkret (.) behilflich sein**
(2.6)
KL1 hm
(2.1)
KL1 also zum einen
(1.9)
KL1 vielleich
(0.5)
KL1 haben sie ja irgendwie durch fragen oder so die möglichkeit
bisschen klarheit in meine gedank[en] .h ähm (.) zu fassen

Example 5: Requesting Agenda Agreement

1 CO7 **wär das hier ein moment in unsrem gespräch was vielleicht für sie auch nützlich**
2 **wär mal zurückzuschaun was sie über sich und das thema vielleicht schon so**
3 (0.5)
4 CO7 **rausgefundn habn**
5 (2.2)
6 KL1 ja
7 (0.3)
8 KL1 s_kömma gerne machen

Example 6: Requesting Agenda Action

1 CO1 wenn ma schnell bereit is .hh gut ((schnalzt)) .h ähm hhh ja jetzt äh
2 si äh sim ma (ham ma) noch mal so_n bisschen umkreist äh wo
3 wie wie so ihre sachlage .h is .h etz würd ich aber doch ganz gerne
4 noch mal .h ein bisschen präzisieren was wir heute .h machen können
5 .h ich hab mir noch mal (.) meine notizen durchgeschaut .h ((schnalzt))
6 und da kamen mir so .h
7 (0.3)
8 CO1 zwei wichtige punkte .hh was sie gsagt ham und **da korrigieren sie mich**
9 **bitte auch [wieder] wenn ich mir des falsch zusammengefasst hab** .hh
10 KL1 [hmhm]
11 CO1 also so zwei anli liegen gewissermaßen in diesem coaching .hh
12 dass sie also mal ganz generell sie erwarten über_s coaching
13 mal en perspektivenwechsel
14 (0.2)
15 [so f]ür sich selbst .hh ähm (.) in ihren handlungen
16 KL1 [hmhm]
17 CO1 oder ihrer be (.) selbstbetrachtung .h und

Example 7: Suggesting Agenda Action

CO1 .h ((schnalzt)) (.) das is so [das was hhh ich]
KL1 [((unverständlich)), lacht))]
CO1 mir jetz so (.) .hh [not]iert hab (.) .h
KL1 [ja]
(0.6)
CO1 ähm (.)sie finden da unten auch noch so erläuterung **.h sie können ja auch**
noch .h (.) leute die ihnen nahe stehen oder auch sei_s kollegen sei_s (.)
ihren mann oder s[o mal fr]agen .h einfach nur mal so
KL1 [hmhm]
CO1 ihre aufgabe wäre sozu[sagen] .h vornehm zuhören hhh
KL1 [ja]
(0.4)
KL1 ja
(0.6)
CO1 also und dann einfach mal so du was siehst_n du was ich besonders gut kann
und so welche fähigkeiten (.) .h siehst du denn bei mir

Example 8: Offering Agenda Action

1 KL2 ja irgendwie dann (.) jetz auch grad zur sommerzeit dann .h gehen viele raus
2 und machen was schönes dann denk ich immer so un jetz hock ich hier zu hause .h
3 CO3 hmhm
4 KL2 hab ich_s gefühl ich hab gar kein lebensgefühl dann dann nervt mich das
5 CO3 versteh ich gut
6 (0.2)
7 KL2 und äh dann bin ich irgendwie depri[miert]
8 CO3 [ja]
9 KL2 und denke alle ham n leben und ich hab gar kei[ns]
10 CO3 [ah]
11 CO3 **ha ha ha [.hh](.) öhm okay aber darf ich ihnen da ein[fach so] so_n**
12 KL2 [ja] [ja]
13 CO3 **paar tipps so [ausm] zeit und aufgabenmanagement geben** .hh öhm (.)
14 KL2 [ja]
15 CO3 äh das erste was ich ihnen da empfehle is halt dass sie wenn sie solche
16 arbeits(.)slots für sich einplanen ja .hh äh wenn sie fühlen es geht gar nicht
17 dass sie_s dann auch sein la[ssen da]ss sie sich selber erlauben heute geht_s
18 KL2 [hm]
19 nicht ich merke (.) ich ich brauch eher genau wie die anderen meine freizeit ich
20 muss jetz ma spazieren gehen

Example 9: Proposing Agenda Action

(0.2)
CO1 ((schnauft)) okay .h und (.)wenn sie sich jetz so zeit für sich nehmen .h
dann hätt ich da gleich noch was was ich ihnen .h (.) jenseits von den
zeit tools [.hh]des is a sach das lesen se sich schnell du[rch .h]
KL1 [hmhm] [ja]
CO1 und dann schaun_s amal einmal ausprobieren
and **und dann kö_ma äh auch noch mal so kurz abstimmen** .h
(0.2)
CO1 ((schnalzt))
(0.4)
CO1 is es
(0.3)
CO1 stimmig irgendwie passt_s oder äh wo ha wo
(0.2)
CO1 wo is es en bisschen holprig .h des andere is .h
es geht ja um ihre ressourcen .h
KL1 ja
